# Implementing custom PRO and NPS tools in private practice: balancing simplicity and clinical value in a retrospective study

**DOI:** 10.1186/s41687-026-01110-4

**Published:** 2026-06-09

**Authors:** Paul Bonilla, Juan Pablo Flanagan, Anesu Murambadoro, Victoria Elizondo, Michael Sander

**Affiliations:** 1 University of Texas Rio Grande Valley School of Medicine, 1201 West University Drive, EMEBL 1.117, Edinburg, 78539-2909 TX UK; 2 Ponce Health Sciences University School of Medicine, PO Box 7004, Ponce, PR 00732 USA

**Keywords:** Patient reported outcomes, Private practice, Three c’s, Reimbursement, Functionality, Postsurgical pain

## Abstract

**Background:**

The authors describe the development of a custom survey centered around the Three C’s and Net Promoter Scores in a high-volume surgical private practice with limited resources and no research coordinator. This paper aims to examine whether an abbreviated survey can serve as a practical alternative to lengthy validated PROMs in tracking post-surgical recovery outcomes in a private practice setting.

**Methods:**

A retrospective, de-identified analysis was conducted on prospectively collected patient-reported outcome surveys administered via email preoperatively and 120-days postoperatively. Of 2,908 surveys administered, 101 patients undergoing four high-volume procedures (CTR, meniscectomy, TKR, hernia) with matched pre- and post-operative responses were included. Paired t-tests with Shapiro-Wilk normality testing were performed in R (version 4.3.2) at α = 0.001.

**Results:**

In total, 2,908 surveys were administered between 2021 and 2024 with 719 pre-surgery responses collected and 439 responses to both pre- and post-surgery surveys. The highest volume cases were selected for analysis based on their high surgical volume (> 8 cases), clinical relevance to orthopedics, and their frequent association with post operative recovery function. Surgical patients undergoing Carpal Tunnel Release (CTR) (t = 4.04, *p* < 0.001), Meniscectomy (t = 3.46, *p* < 0.001), Total Knee Replacement (TKR) (t = 4.15, *p* < 0.001), and Total Hip Replacement (THR) (t = 4.71, *p* < 0.001) demonstrated significant improvements in post-surgical numerical pain score. Similar results were observed in functionality and disruptive stress scores. Researchers observed a calculated NPS of 88.2 of which 400 were promoters, 26 were passives, and 13 were detractors.

**Conclusion:**

The authors present this work as an initial signal study demonstrating that a brief and self-administered survey can capture statistically significant improvements in pain, functional difficulty, and psychological stress in a private practice setting with limited research infrastructure. While NPS and work metrics should not be used alone as a gauge of outcomes, they hold significant utility in conjunction with other patient reported outcomes. Reporting requirements by the Center for Medicare and Medicaid Services (CMS) require hospitals to collect pre surgery and one year post surgery (9–15 months) HOOS JR and KOOS JR scores for total joint replacement surgeries, with public reporting set for 2027 and reimbursement effects in 2028. These requirements underscore the need for streamlined solutions. As value-based care becomes a priority, practical strategies are needed to reduce the burden on providers and patients [1].

## Background

Patient reported outcome measures (PROMs) are standardized, validated questionnaires completed by patients to assess health status, symptoms, and functional outcomes but are distinct from patient reported experience measures (PREMs) which capture subjective experiences of care processes and interactions [2]. Patient-reported outcomes (PROs) are essential for tracking patient progress, but lengthy validated surveys, such as the 40 question Knee Injury and Osteoarthritis Outcome Score (KOOS), create time and costly challenges for providers, especially in private practice [3, 4]. A national survey of hand surgeons found that private practice surgeons were significantly less likely to collect PROMs compared to their academic counterparts due to the administrative burden, cost, and lack of infrastructure cited as the primary barriers [5]. A multi-institutional survey of surgeons and care teams identified staff burden, data infrastructure limitations, and lack of reimbursement as the most frequently reported barriers to PRO collection and use which highlights the need for streamlined and low burden alternatives [6]. Starting July 2024, the Centers for Medicare and Medicaid (CMS) now require hospitals to collect pre surgery and one year post surgery (9–15 months) HOOS Joint Replacement and KOOS Joint Replacement scores for total replacement surgeries, with public reporting set for 2027 and reimbursement effects in 2028 [7]. The recent analyses of PRO performance measures within CMS alternative payment models have identified abbreviated collection tools as critical enablers of widespread adoption in resource limited practices [8]. These financial penalties underscore the need for streamlined solutions. As value-based care becomes a priority, practical strategies are needed to reduce the burden on providers and patients. The *Three C’s* framework offers a functional approach. Liu et al. define *Capability* as the measure of a patient’s functional status or difficulties in activities of daily living (ADL), *Comfort* as measuring relief from physical and emotional pain, and *Calm* as measuring the extent to which patients can continue their life during care by considering elements like how many hours per week the patient spends scheduling and traveling to appointments [9]. This framework encompasses improvements from treatment but also addresses the burden of suffering, disruptions of life, and the stress of receiving healthcare [9].

Another metric that has gained popularity in assessing patient satisfaction across industries is the Net Promoter Score (NPS). This score was introduced by Reichheld in 2003 as a patient experience measure related to but distinct from formal PREMs [10]. While PREMs typically capture multi-dimensional experiences across care touchpoints, NPS distills the patient experience into a single recommendation likelihood score, offering a practical and easily interpretable alternative for busy clinical settings [2, 10]. This widely used tool measures customer experience and loyalty through a simple question: “How likely are you to recommend [organization name] to a friend or colleague?” Responses categorize individuals as Promoters (9–10), Passives (7–8), or Detractors (0–6), with the final score calculated by subtracting the percentage of Detractors from Promoters. Introduced in 2003, NPS has become popular for its simplicity, predictive value, and benchmarking capabilities. In healthcare, NPS adoption is increasing as hospitals seek concise, standardized tools to assess patient satisfaction—an essential metric in the shift toward value-based care. By providing an easily interpretable score, NPS offers a practical way to emphasize patient-centered outcomes, enabling physicians to improve patient experiences. This study evaluates the utility of NPS as a tool for assessing satisfaction in surgical patients, where accurate and actionable feedback is critical.

Managing patient expectations before surgery is essential to improving the surgical experience. A key aspect of this is understanding the impact of surgery on work. While patient-reported outcomes (PROs) are commonly used to assess health conditions, less attention has been given to how work performance, both before and after surgery, can inform preoperative counseling. Analyzing the impact of surgery on work can reveal patterns that help patients make informed decisions, improve long-term outcomes, and enhance satisfaction by setting achievable recovery timelines.

This study presents the preliminary implementation of a shorter, focused survey in private practice based on the *Three C’s*, ease of work, and NPS for a practical alternative in tracking post-surgical recovery in a resource limited setting and no research coordinator.

## Methods

Patient-reported outcomes measurements were collected by a private practice surgery provider. Surveys were administered preoperatively and 120-days postoperatively. Surveys were administered electronically using SurveySparrow which enabled automatic scheduling and delivery of pre-operative and 120 day post operative surveys via email to all clinic patients. This automatic delivery system eliminated the need for manual survey distribution and reducing administrative burden on clinical staff. Questions employed a 10-point scale to measure numerical rating scores (NRS) for pain, functional status in activities of daily living, disruptive stress levels, and ease of work. Survey questions were developed by the practice team with input from Dell Medical School faculty based on the Three C’s framework and were adapted from validated instruments to ensure alignment with recognized patient reported outcome domains (Table 1) [9]. In total, 2,908 surveys were administered, yielding 719 preoperative responses and 439 matched pre-and post-operative responses. Of these 439 patients, 101 undergoing specific high-volume orthopedic and general surgery procedures (volume ≥ 8) were included in the primary subgroup analysis: carpal tunnel release (CTR) (*n* = 20), meniscectomy (*n* = 23), total knee replacement (TKR) (*n* = 14), and hernia surgeries (*n* = 36). Analysis was performed in R (version 4.3.2), which included box plots and descriptive statistics. 120-day postoperative surveys also included an NPS question. Preoperative and postoperative pain scales were compared using paired t-tests to investigate statistically significant differences between pre- and post-operative scores. Prior to analysis, normality was assessed visually using histograms and Q-Q plots, formally evaluated using Shapiro-Wilk (SW) test to determine whether differences between pre and post scores were normally distributed. A significance level of α = 0.001 was applied to all analyses. Standard deviations (SD) and interquartile ranges (IQR) were reported alongside means.

**Table 1:** Survey questions presented to patients

Survey Questions	Minimum	Maximum
Comfort: To what degree do you experience pain during daily activities?	*0: No Pain*	*10: Worst Pain Imaginable*
Capability: Please indicate the degree of difficulty you have experienced in the last week due to your condition	*0: No Difficulty*	*10: Extreme Difficulty*
Calm: To what degree do you feel managing your stress or handling the logistics of your condition interferes with your daily life?	*0: No Disruption*	*10: Extreme Disruption*
Ease of Work: To what degree are you able to work while experiencing your condition?	*0: Unable to Work*	*10: Able to Work*
NPS: How likely are you to recommend [Company Name] to friends and family?	*0*	*10*

NPS, Net Promoter Score

## Results

A total of 101 patients were included in the primary subgroup analysis across four high volume procedures: hernia repair (*n* = 36), CTR (*n* = 20), meniscectomy (*n* = 23), and total knee replacement (*n* = 14), and total hip replacement (*n* = 8). Pre-operative scores across all surgery types reflected moderate to severe symptom burden with mean pain scores ranging from 4.4 in the hernia cohort to 8.0 in the hip replacement cohort, consistent with clinical severity expected in patients presenting for surgical intervention. Post-operative scores demonstrated consistent reductions across all three domains (Table 2).

**Table 2:** Three c scores by surgery type

Surgery Type	*N*	Metric	t-statistic	*p*-value	Pre Survey Mean (SD)	Post Survey Mean (SD)	Pre Survey IQR	Post Survey IQR	SW *p*-value
Hernia	36	Pain	3.129	*p* < 0.004	4.4 (2.5)	3.0 (2.3)	4.3	3.0	0.096
		Difficulty	5.372	*p* < 0.001	4.3 (2.8)	1.8 (1.6)	5.0	1.0	0.001*
		Stress	5.152	*p* < 0.001	5.1 (3.2)	2.3 (2.2)	6.0	1.0	0.001*
Carpal Tunnel Release (CTR)	20	Pain	4.043	*p* < 0.001	7.1 (2.5)	3.7 (2.9)	2.3	4.3	0.484
		Difficulty	5.250	*p* < 0.001	7.2 (3.0)	3.0 (2.9)	5.0	2.5	0.169
		Stress	4.351	*p* < 0.001	5.6 (3.3)	2.2 (2.1)	4.8	1.3	0.022*
Knee Meniscectomy	23	Pain	3.456	*p* = 0.002	6.0 (2.4)	3.9 (2.5)	3.5	4.0	0.537
		Difficulty	6.308	*p* < 0.001	6.0 (2.0)	2.5 (1.7)	3.5	3.0	0.052
		Stress	6.223	*p* < 0.001	6.7 (2.5)	2.9 (2.2)	4.0	4.0	0.541
Total Knee Replacement (TKR)	14	Pain	4.153	*p* = 0.001	7.2 (1.9)	3.1 (2.6)	3.0	1.0	0.039*
		Difficulty	4.178	*p* = 0.001	7.2 (1.5)	3.3 (3.4)	2.8	2.0	0.023*
		Stress	5.196	*p* < 0.001	5.8 (3.5)	1.6 (1.2)	6.8	1.0	0.352
Total Hip Replacement (THR)	8	Pain	4.714	*p* = 0.002	8.0 (0.5)	3.9 (2.7)	0.0	3.5	0.593
		Difficulty	8.275	*p* < 0.001	8.3 (0.9)	2.6 (1.8)	1.3	2.3	0.603
		Stress	5.481	*p* < 0.001	7.8 (2.2)	3.1 (2.5)	1.5	4.3	0.203

N, number; SD, standard deviation; IQR, Interquartile range; SW, Shapiro-Wilk; *, non-normal

### Three C’s

**Fig. 1:**
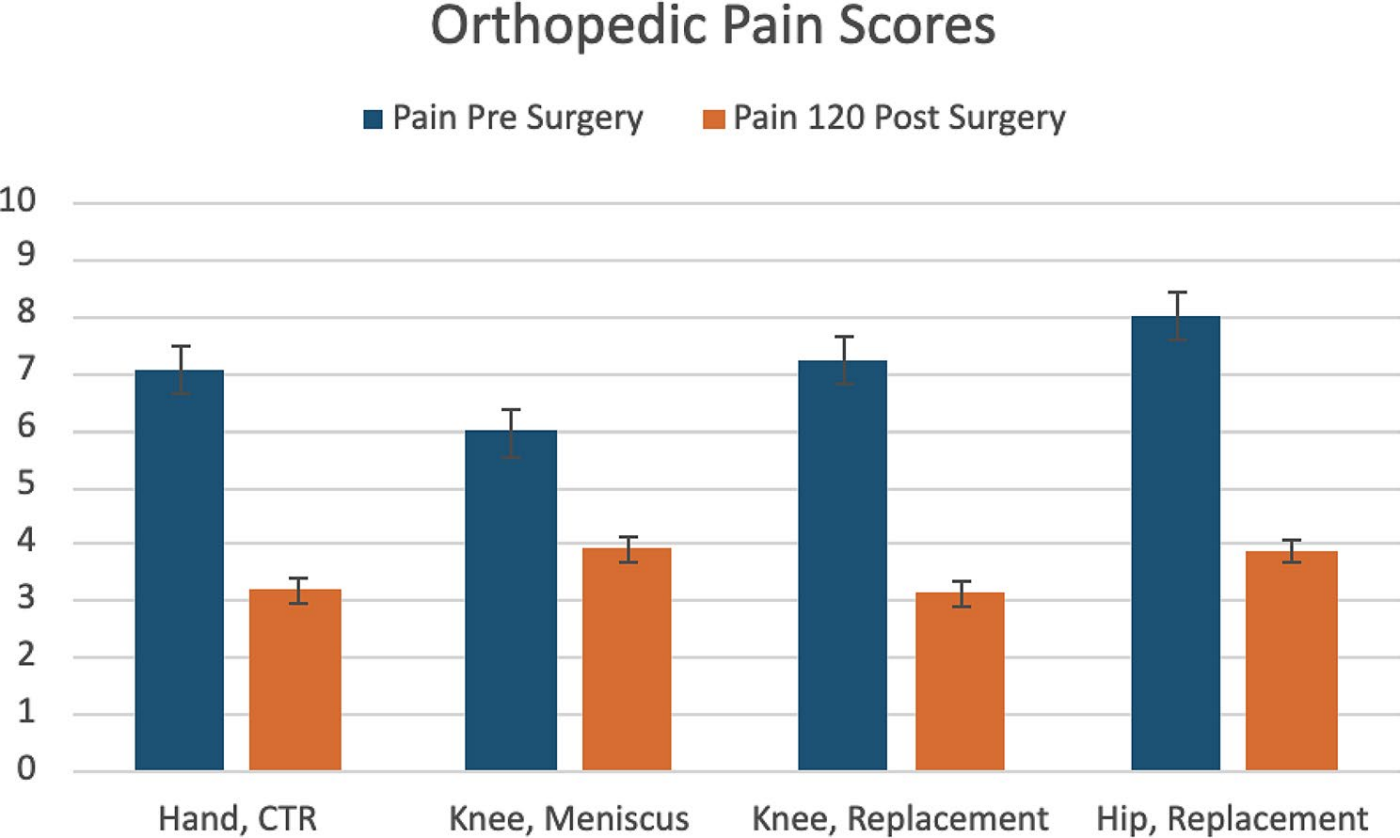
Pain scores by surgery type

**Fig. 2:**
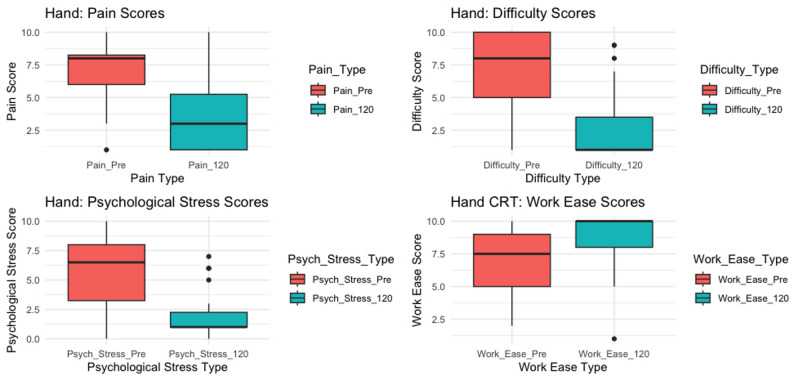
Carpal tunnel scores

**Fig. 3:**
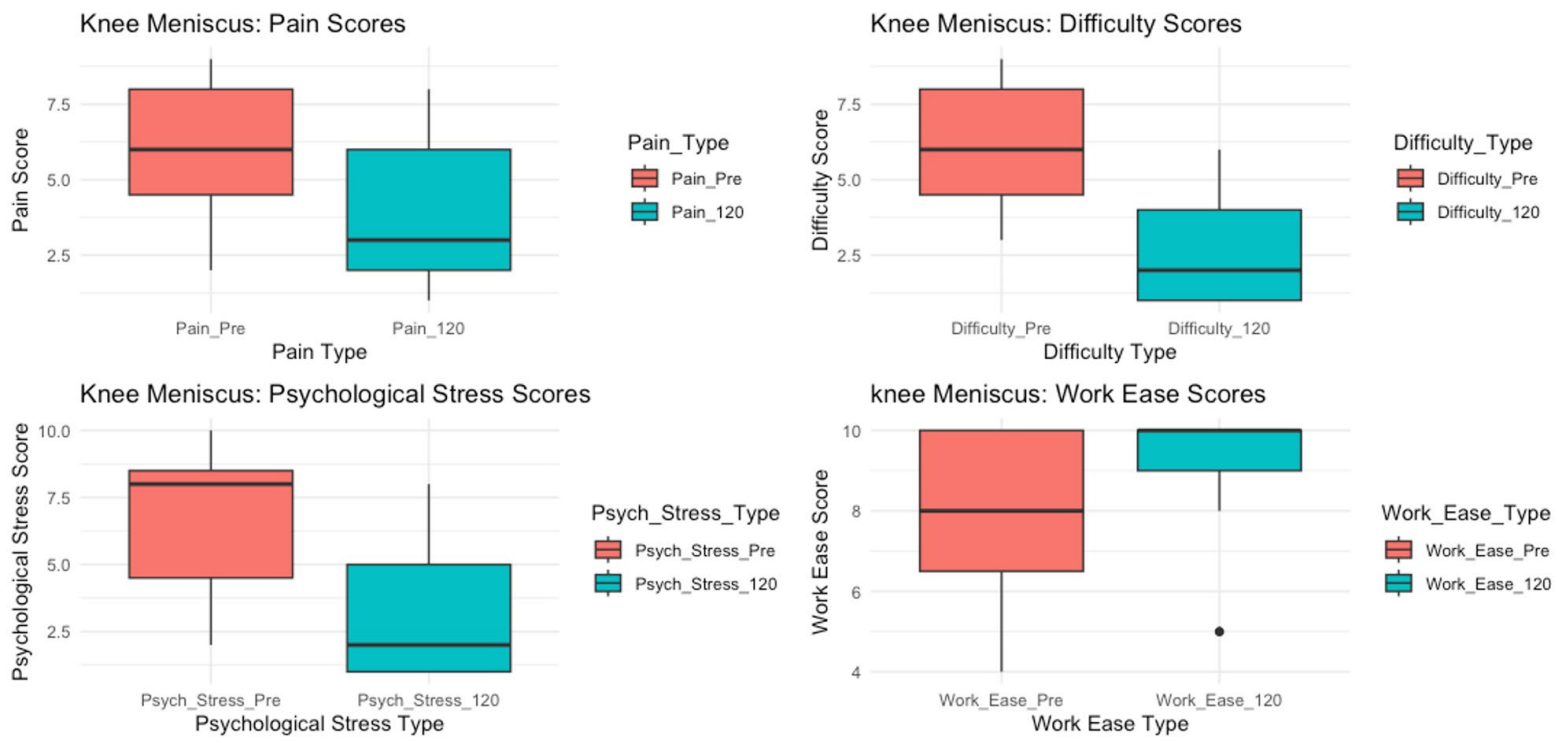
Meniscectomy scores

**Fig. 4:**
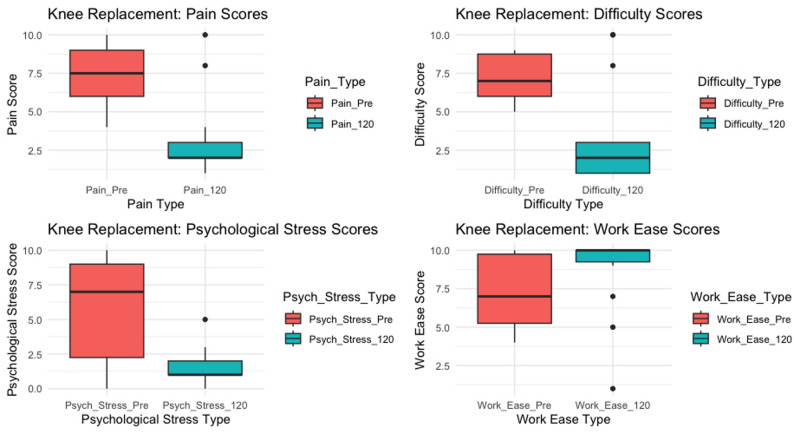
Total knee replacement scores

CTR mean pain scores decreased from 7.1 (median = 8.0) to 3.7 (median = 3.0) (Table 2). Similar results were observed for functionality (mean: 7.2 to 3.0) and stress (mean: 5.6 to 2.2). Paired t-tests were used to evaluate significance. CTR demonstrated significant improvements post-surgery: pain (t = 4.04, *p* < 0.001), functionality (t = 5.25, *p* < 0.001), and stress (t = 4.35, *p* < 0.001). Between surgery types, patients undergoing joint replacement demonstrated the most notable reduction in pain NRS (Fig. 1). Patients also demonstrated median improvements in the ease of their work post-surgery (Figs. 2 and 3, and Fig. 4).

Patients undergoing meniscectomy demonstrated a 34% reduction in mean pain scores. Similar results were observed for functionality (mean: 6.0 to 2.5) and stress (mean: 6.7 to 2.9) (Table 2). Significant improvements were also noted post-surgery: pain (t = 3.46, *p* = 0.002), functionality (t = 6.31, *p* < 0.001), and stress (t = 6.22, *p* < 0.001).

Patients undergoing TKR demonstrated a 56% reduction in mean pain scores. Similar results were observed for functionality (mean: 6.0 to 2.5) and stress (mean: 6.7 to 2.9) (Table 2). Significant improvements were also noted post-surgery: pain (t = 4.15, *p* = 0.001), functionality (t = 4.18, *p* < 0.001), and stress (t = 5.20, *p* < 0.001).

### NPS

**Fig. 5:**
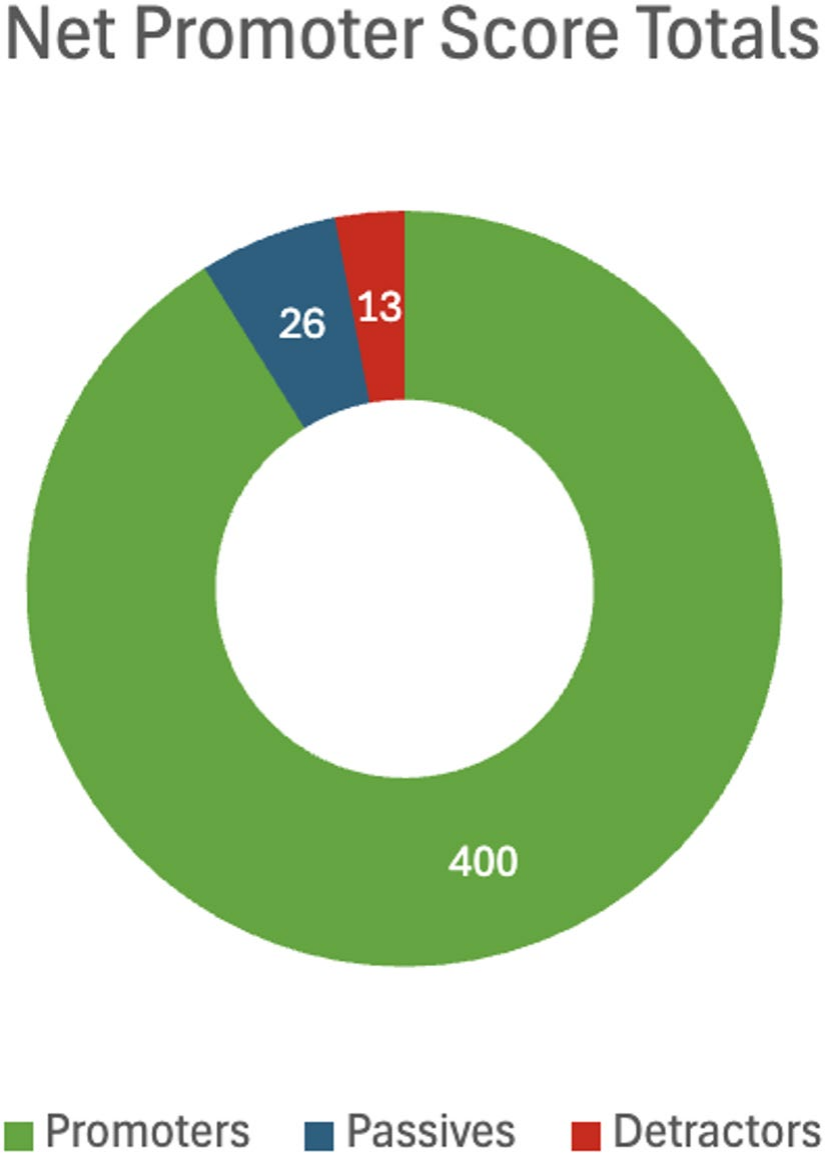
NPS totals

Researchers observed a calculated NPS of 88.2 of which 400 were promoters, 26 were passives, and 13 were detractors (Fig. 5). In the NPS framework, Promoters (9–10) are considered loyal enthusiastic patients that are likely to recommend the practice, Passives (score 7–8) are satisfied but unenthusiastic, and Detractors (score 0–6) are considered dissatisfied patients who do not see your service as something worth recommending to friends and family [10, 11]. The NPS is calculated by subtracting the percentage of Detractors from the percentage of Promoters, yielding a score ranging from − 100 to + 100. An overall of NPS of 88.2 compares favorably with published benchmarks for healthcare organizations where average scores range from 30 to 58 depending on specialty and setting [11]. CTR patients demonstrated a NPS of 100 (*n* = 20), hernia patients demonstrated a NPS of 100 (*n* = 36, promoters: 31, passives: 5), and meniscectomy patients demonstrated a NPS of 100 (*n* = 23). Interestingly, TKR patients demonstrated a NPS of 71 (*n* = 14: passives: 12, detractors: 2).

## Discussion

This study demonstrates that concise patient-reported outcome surveys may effectively capture meaningful clinical improvements following common outpatient surgical procedures. Statistically significant improvements were observed across every surgery type analyzed. For example, CTR patients experienced a 47.9% reduction in pain scores (mean: 7.1 to 3.7, *p* < 0.001), a 58.3% improvement in functional difficulty (mean: 7.2 to 3.0, *p* < 0.001), and a 60.7% decrease in stress levels (mean: 5.6 to 2.2, *p* < 0.001). Similar trends were seen in meniscectomy patients, with pain decreasing from 6.0 to 3.9 (*p* = 0.002), difficulty from 6.0 to 2.5 (*p* < 0.001), and stress from 6.7 to 2.9 (*p* < 0.001).

TKR patients also demonstrated marked improvements: pain dropped from a mean of 7.2 to 3.1 (*p* = 0.001), functionality improved from 7.2 to 3.3 (*p* = 0.001), and stress fell from 5.8 to 1.6 (*p* < 0.001). These findings suggest that even brief, focused instruments may detect changes in physical and emotional well-being, supporting their potential as efficient tools in clinical practice [12]. Patient-reported outcome data, when fed back to clinicians in a timely and structured manner, have been shown to improve identification of unresolved symptoms, enhance shared-decision making, and drive quality improvement in outpatient surgical practice [13, 14]. Expanding the collection of work-related metrics such as ease of work and days of missed work may offer providers a holistic understanding of treatment success and guide surgical decision making. Standard deviations and IQRs reported in Table 2 reveal notable variability, particularly in the Hip Replacement cohort where pre-operative pain SD was only 0.5, indicating a homogeneous, high severity patient population and likely due to the small sample size (*n* = 8). Shapiro-Wilk testing of pre-post difference scores identified five surgery combinations with non-normal distributions (marked * in Table 2), most notably in Hernia Difficulty, Hernia Stress, CTR Stress, TKR Pain, and TKR Difficulty. These findings should be noted as a limitation of the parametric approach used. The authors present this work as an initial signal study demonstrating that a brief and self-administered survey can capture statistically significant improvements in pain, functional difficulty, and psychological stress in a private practice setting with limited research infrastructure.

Patients today have access to abundant healthcare information, allowing them to be more selective in choosing providers. Business economics teaches that healthy competition results in process improvement and lower costs, while noncompetitive providers are driven out of the market [15]. Health care exhibits a nontraditional buyer’s market, in which providers cannot be compared as when purchasing a car. Although patient health varies, objective measures of results and complications by disease will assist the customer in choosing a provider in the future, but this level of information is currently unavailable [15]. Consumer reviews through NPS may offer a solution to bridge that gap. An overall NPS of 88.2 in this study indicates a high level of satisfaction among patients [10, 11]. Notably, procedures such as CTR, hernia repairs, and meniscectomies all yielded perfect NPS scores of 100, suggesting exceptional patient experience. However, TKR patients reported a lower NPS of 71, with 12 passives and 2 detractors among 14 respondents. This discrepancy may highlight a misalignment between clinical outcomes and patient expectations, emphasizing the importance of managing expectations for postoperative recovery in more complex procedures. Other studies have observed similarities in differences between procedures, with one study observing an NPS of 71 for THR and 49 for TKR [11]. A different study demonstrated promoters always showed significantly better outcomes than detractors [16].

While NPS should not be used alone as a gauge of outcomes, it holds significant utility in conjunction with other patient reported outcomes. It is also important to acknowledge the controversial nature of patient satisfaction as a healthcare quality metric. Optimizing NPS or satisfaction scores can create perverse incentives for providers in which they avoid disclosing unfavorable prognostic information or withhold necessary but unwelcome recommendations if doing so might negatively impact satisfaction ratings. This dynamic can undermine shared decision making and patient health literacy. The authors recognize that NPS should be interpreted cautiously and never used as the sole determinant of care quality of provider performance. Rather, it should be considered one of several complimentary metrics, alongside clinical outcomes and validated patient reported measures. Research has shown that optimizing satisfaction metrics can lead to increased healthcare expenditures and may not correlate with improved health outcomes [5].

These results support the feasibility of deploying shorter surveys in healthcare, where efficiency and patient satisfaction are paramount. Shorter surveys are especially valuable in outpatient settings, where resource limitations must be factored. These shortened tools are adaptable and could be integrated into hospital systems to reduce burden while capturing meaningful data that matters to patients. Email delivery of PRO surveys has been shown to significantly improve response rates and reduce administrative burden compared to paper-based models [17].

Compared to lengthy instruments like KOOS or HOOS which exceed 40 questions, the use of these focused questions plus NPS offers a practical solution with minimal burden. However, it is important to acknowledge the advantages of validated PROMs and PREMs as these tools have undergone rigorous psychometric testing to establish their validity, reliability, and responsiveness to clinical change. In contrast, the survey used in this study has not yet been formally validated and therefore its measurement properties remain uncertain. While the brevity and ease of administration of our tool are practical strengths, they should not be interpreted as substitutes for validated instruments which provide defensible data for clinical decision making and research. Validated PROMs and PREMs provide established psychometric properties including test-retest reliability, construct validity, and responsiveness to change, properties that the present survey has not yet demonstrated [2]. Nevertheless, the study has limitations. The data originate from a single organization, which may limit generalizability. Additionally, smaller sample sizes in some subgroups may affect the robustness of certain statistical comparisons. Furthermore, the short surveys used, while pragmatic, are not yet validated against gold-standard PRO measures such as shorter KOOS JR or Patient-Reported Outcomes Measurement Information System (PROMIS). Recent guidance on PRO instrument development recommends a structured validation pathway encompassing content validity, construct validity, test-retest reliability, and unresponsiveness to clinical change as minimum standards before clinical deployment [18, 19].

Future work should focus on validating these tools against established surveys and exploring their responsiveness to clinical interventions over time. Expanding this model to other specialties and institutions will also help assess its scalability. In the transition toward value-based care, this study adds to the growing evidence that shorter, targeted PROs can maintain analytic rigor while enhancing clinical utility, which may be especially useful in private practice where resources may be limited and there are perceptions of burdens to adoption [20–22]. To formally recommend this abbreviated tool as a clinical alternative, future studies should assess the sensitivity and specificity of these measures relative to validated PROMs. Additionally, if a gold standard is unavailable, a concordance/discordance analyses comparing scores from both instruments in a parallel cohort would offer an estimate of agreement and meaningfully increase the clinical impact of this research. Expanding PRO collection to broader musculoskeletal populations would allow assessment of whether these abbreviated tools demonstrate the construct validity and responsiveness observed in longer validated instruments across different surgical specialties [13]. Research has demonstrated that electronic PRO platforms can substantially reduce this burden when integrated into existing workflow systems, although information technology infrastructure remains a key implementation challenge in ambulatory and private practice settings [23]. By pairing symptom tracking with simple and interpretable satisfaction metrics, healthcare systems can better align care delivery with patient needs and expectations. A key limitation of the study is the absence of a validated survey instrument as an appropriate control. The use of a KOOS JR or PROMIS control could confirm the observed improvements in pain and functionality. Additionally, Pearson or Spearman’s correlations between our survey scores and established instruments mentioned could be administered to predictive validity while Cronbach’s alpha could be used for internal consistency. Critically, shorter questionnaires involve an inherent tradeoff between measurement specificity and patient burden.

## Conclusion

While the surveys used in this study were not nationally recognized, they provide a promising step toward more efficient tools for measuring outcomes. Significant improvements in pain, functionality, and stress were documented, suggesting these shorter surveys may serve as a useful tool for tracking recovery. To further validate their effectiveness, future studies should compare the short survey results with established PROs, assess test-retest reliability, and measure responsiveness to clinical changes. Patient and provider feedback will also ensure these tools are practical and meaningful in real-world settings. Mandatory reporting and financial penalties for non-compliance by the CMS will have significant implications for the future of healthcare in the United States. As payers increasingly tie reimbursement to PRO-based performance measures in the coming years, the development of practical, abbreviated, and electronically administered tools that meet both clinical and regulatory standards will be essential for private practices seeking to remain competitive in value based care models [8, 23].

Identifying areas of patient dissatisfaction allows organizations to address potential gaps in care. For example, lower scores in knee replacement surgeries could highlight the need for better pain management protocols. Transparency is essential in healthcare to foster trust and ensure high quality care. Recent legislation mandates the public reporting of Hip Disability and Osteoarthritis Outcomes Score for Joint Replacement (HOOS JR) and Knee injury and Osteoarthritis Outcome Score for Joint Replacement (KOOS JR), however, these metrics may be challenging to fully grasp for patients with low health literacy. The NPS score, however, offers a straightforward numerical value. As the transition to value-based care continues, standardized tools like NPS can streamline patient satisfaction measurement, enabling comparisons across providers and institutions. By adopting NPS, healthcare systems can enhance quality assurance and prioritize patient-centered care, ultimately optimizing the healthcare experience. Although the study’s reliance on data from a single organization and smaller sample sizes may limit the generalizability of the findings, the potential of NPS as a tool for improving patient care remains promising.

## Data Availability

The datasets used and/or analyzed during the current study are available from the corresponding author on reasonable request.

## Abbreviations

PRO: Patient Reported Outcomes
PROM: Patient Reported Outcome Measures
PREM: Patient Reported Experience Measures
ADL: Activities of Daily Living
NRS: Numerical Rating Score
TKR: Total Knee Replacement
THR: Total Hip Replacement
CTR: Carpal Tunnel Release
CMS: Center for Medicare and Medicaid Services
KOOS: Knee Injury and Osteoarthritis Outcome Score
HOOS: Knee Injury and Osteoarthritis Outcome Score
NPS: Net Promoter Score
PROMIS: Patient-Reported Outcomes Measurement Information System

## References

[1] Porter ME, Lee TH (2016) From volume to value in health care: The work begins. JAMA - J Am Med Association 316(10). 10.1001/jama.2016.11698 10.1001/jama.2016.11698 27623459

[2] Kingsley C, Patel S (2017) Patient-reported outcome measures and patient-reported experience measures. BJA Educ 17(4). 10.1093/bjaed/mkw060

[3] Lavallee DC, Chenok KE, Love RM et al (2016) Incorporating patient-reported outcomes into health care to engage patients and enhance care. Health Aff 35(4):575–582. 10.1377/hlthaff.2015.1362 10.1377/hlthaff.2015.1362 27044954

[4] Roos EM, Lohmander LS (2003) The Knee injury and Osteoarthritis Outcome Score (KOOS): From joint injury to osteoarthritis. Health Qual Life Outcomes 1. 10.1186/1477-7525-1-64 10.1186/1477-7525-1-64 PMC280702 14613558

[5] Fenton JJ, Jerant AF, Bertakis KD, Franks P (2012) The cost of satisfaction: A national study of patient satisfaction, health care utilization, expenditures, and mortality. Arch Intern Med 172(5). 10.1001/archinternmed.2011.1662 10.1001/archinternmed.2011.1662 22331982

[6] Snyder DJ, Park C, Ahn A, et al (2021) Barriers to collection and use of patient-reported outcomes a multi-institutional survey of surgeons and care teams. Bull Hosp Joint Dis 79(3) 34605754

[7] Franco JR, Chen AF, Ready JE et al (2025) The CCJR^®^ Gerard A. Engh excellence in knee research award: patient-reported outcomes collection and mandatory medicare inpatient total knee arthroplasty patient-reported outcome performance measures: how to optimize the process. J Arthro. Published online. 10.1016/j.arth.2025.03.033 10.1016/j.arth.2025.03.033 40120654

[8] Gettel CJ, Suter LG, Bagshaw K et al (2024) Patient-reported outcome-based performance measures in alternative payment models: current use, implementation barriers, and principles to succeed. Value Health 27(2). 10.1016/j.jval.2023.10.017 10.1016/j.jval.2023.10.017 PMC10872237 38042334

[9] Liu TC, Bozic KJ, Teisberg EO (2017) Value-based healthcare: person-centered measurement: focusing on the three C’s. Clin Orthop Relat Res 475(2):315–317. 10.1007/s11999-016-5205-5 27995559 10.1007/s11999-016-5205-5 PMC5213966

[10] Reichheld, Frederick F (2003 Dec) The one number you need to grow. Harv Bus Rev (4) 14712543

[11] Adams C, Walpola R, Schembri AM, Harrison R (2022) The ultimate question? Evaluating the use of Net Promoter Score in healthcare: A systematic review. Health Expectations John Wiley Sons Inc 25(5):2328–2339. 10.1111/hex.13577 10.1111/hex.13577 PMC9615049 35985676

[12] Rotenstein LS, Huckman RS, Wagle NW (2017) Making Patients and Doctors Happier — The Potential of Patient-Reported Outcomes. N Engl J Med 377(14). 10.1056/nejmp1707537 10.1056/NEJMp1707537 28976860

[13] Jayakumar P, Heng M, Okelana B, Vrahas M, Rodriguez-Villalon A, Joeris A (2023) Patient-reported outcome measurement in orthopaedic trauma. J Am Acad Orthop Surg 31(20). 10.5435/JAAOS-D-23-00375 10.5435/JAAOS-D-23-00375 37796280

[14] Greenhalgh J, Dalkin S, Gooding K et al (2017) Functionality and feedback: a realist synthesis of the collation, interpretation and utilisation of patient-reported outcome measures data to improve patient care. Health Serv Delivery Res 5(2). 10.3310/hsdr05020 28121094

[15] Kinney WC (2005) A simple and valuable approach for measuring customer satisfaction. Otolaryngol Head Neck Surg 133(2):169–172. 10.1016/j.otohns.2005.03.060 16087007 10.1016/j.otohns.2005.03.060

[16] Osmanski-Zenk K, Ellenrieder M, Mittelmeier W, Klinder A (2023) Net promoter score: a prospective, single-centre observational study assessing if a single question determined treatment success after primary or revision hip arthroplasty. BMC Musculoskelet Disord 24(1). 10.1186/s12891-023-06981-y 10.1186/s12891-023-06981-y PMC10605956 37891529

[17] Melucci AD, Liu JB, Brajcich BC et al (2022) Scaling and spreading the electronic capture of patient-reported outcomes using a national surgical quality improvement programme: a feasibility study protocol. BMJ Open Qual 11(4). 10.1136/bmjoq-2022-001909 10.1136/bmjoq-2022-001909 PMC9664302 36375858

[18] Kaneyasu T, Hoshino E, Naito M et al (2024) How to select and understand guidelines for patient-reported outcomes: a scoping review of existing guidance. BMC Health Serv Res 24(1). 10.1186/s12913-024-10707-8 10.1186/s12913-024-10707-8 PMC10938752 38481204

[19] Lee MK, Zaniletti I, Larson DR, Lewallen DG, Berry DJ, Maradit Kremers H (2023) Nuts and bolts of patient-reported outcomes in orthopaedics. J Arthroplasty 38(4). 10.1016/j.arth.2022.11.011 10.1016/j.arth.2022.11.011 PMC10010940 36481287

[20] Shapiro LM, Spindler K, Cunningham B, Koh J (2024) Patient-reported outcome measure collection and utilization: a survey of american academy of orthopaedic surgeons members. J Am Acad Orthop Surgeons Lippincott Williams Wilkins 32(3):114–122. 10.5435/JAAOS-D-23-00879 10.5435/JAAOS-D-23-00879 38181401

[21] Nelson EC, Eftimovska E, Lind C, Hager A, Wasson JH, Lindblad S (2015) Patient reported outcome measures in practice. BMJ (Online) 350. 10.1136/bmj.g7818 10.1136/bmj.g7818 25670183

[22] Teisberg E, Wallace S, O’Hara S (2020) Defining and implementing value-based health care: a strategic framework. Acad Med 95(5):682–685. 10.1097/ACM.0000000000003122 10.1097/ACM.0000000000003122 PMC7185050 31833857

[23] Hsiao CJ, Dymek C, Kim B, Russell B (2019) Advancing the use of patient-reported outcomes in practice: understanding challenges, opportunities, and the potential of health information technology. Qual Life Res 28(6). 10.1007/s11136-019-02112-0 10.1007/s11136-019-02112-0 30684149

